# How do mammillary body inputs contribute to anterior thalamic function?

**DOI:** 10.1016/j.neubiorev.2014.07.025

**Published:** 2015-07

**Authors:** Christopher M. Dillingham, Aura Frizzati, Andrew J.D. Nelson, Seralynne D. Vann

**Affiliations:** School of Psychology, Cardiff University, Tower Building, Park Place, Cardiff CF10 3AT, United Kingdom

**Keywords:** Anterior thalamic nucleus, Diencephalon, Gudden's tegmental nuclei, Learning and memory, Mammillary bodies, Mammillothalamic tract

## Abstract

It has long been assumed that the main function of the mammillary bodies is to provide a relay for indirect hippocampal inputs to the anterior thalamic nuclei. Such models afford the mammillary bodies no independent role in memory and overlook the importance of their other, non-hippocampal, inputs. This review focuses on recent advances that herald a new understanding of the importance of the mammillary bodies, and their inputs from the limbic midbrain, for anterior thalamic function. It has become apparent that the mammillary bodies’ contribution to memory is not dependent on afferents from the subicular complex. Rather, the ventral tegmental nucleus of Gudden is a vital source of inputs that support memory processes within the medial mammillary bodies. In parallel, the lateral mammillary bodies, via their connections with the dorsal tegmental nucleus of Gudden, are critical for generating head-direction signals. These two parallel, but distinct, information streams converge on the anterior thalamic nuclei and support different aspects of spatial memory.

## Highlights

• MB contributions to ATN function are independent of inputs from the hippocampus.
• Inputs from the ventral tegmental nucleus are vital for medial MB function.
• Dorsal tegmental nucleus → lateral MB pathway generates the head-direction signal.
• Lateral and medial MB make parallel but distinct contributions to ATN function.
• MB-ATN projections are critical for recollective but not recognition memory.

## 1 Introduction

**Fig. 1:**
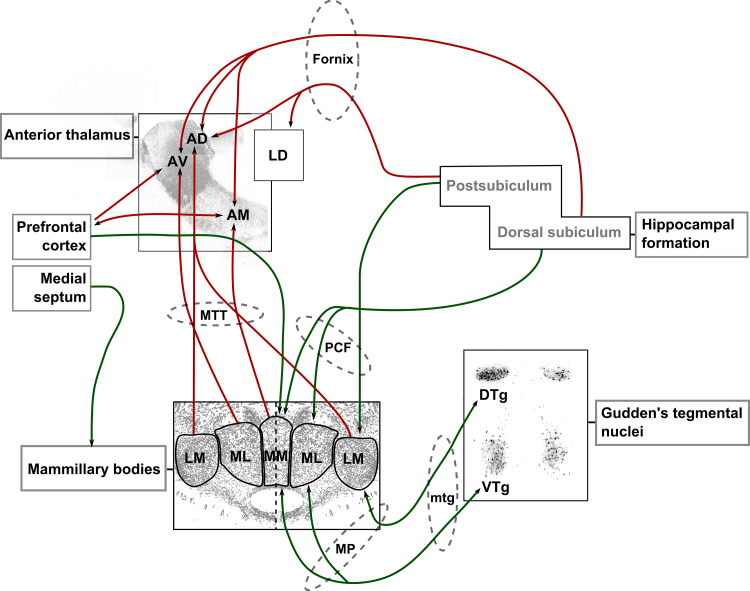
A Semi-schematic diagram showing the major afferent and efferent connections of the mammillary bodies. Mammillary body inputs are represented by green arrows: The medial mammillary nuclei, comprising pars medialis (MM) and pars lateralis (ML) subdivisions, receive input from the dorsal subiculum (via the descending postcommisural fornix (dPCF)) and prefrontal cortex, and have reciprocal connections with the ventral tegmental nuclei of Gudden (VTG), via the mammillary peduncle (mp; VTg/DTG → mammillary bodies) and the mammillotegmental tract (mtg; mammillary bodies → VTG/DTG). The lateral mammillary nuclei are innervated by the postsubiculum and the dorsal tegmental nuclei of Gudden (DTG) via the same respective pathways. In addition, both medial and lateral mammillary body nuclei receive inputs from the medial septum; Anterior thalamic nuclei inputs are represented by red arrows: The major efferent projection of the mammillary bodies is to the anterior thalamic nuclei, via the mammillothalamic tract (MTT). Anterodorsal (AD) and laterodorsal (LD) thalamic nuclei both receive postsubicular inputs while the dorsal subiculum projects to the anteroventral (AV) and anteromedial (AM) thalamic nuclei, all of which are largely via the fornix. In turn, AM has reciprocal connections with the prefrontal cortex. (For interpretation of the references to color in this figure legend, the reader is referred to the web version of this article.)

The anterior thalamic nuclei, a core component of Papez’ circuit, are assumed to form a vital node within a network of related structures that support memory and cognition. Evidence for this assertion comes from the finding that damage or disconnection of the anterior thalamic nuclei is consistently associated with anterograde amnesia in humans and profound learning and memory impairments in rodents (e.g. Aggleton and Sahgal, 1993; Aggleton and Brown, 1999; Carlesimo et al., 2011; Harding et al., 2000; Jankowski et al., 2013). The anterior thalamic nuclei receive inputs (often reciprocal) from a complex array of cortical and subcortical structures; as such, understanding the importance of this circuitry represents a vital step towards uncovering anterior thalamic nuclei functions. Given the extensive direct and indirect hippocampal-anterior thalamic interconnections, as well as the undeniable importance of the hippocampus itself for memory, it is perhaps no surprise that there has been particular focus on the significance of the projections from the hippocampus, via the fornix, for anterior thalamic function (e.g. Aggleton and Brown, 1999). Dense inputs to the anterior thalamic nuclei also arise from the mammillary bodies, reaching the anterior thalamus via the mammillothalamic tract (Cruce, 1975; Seki and Zyo, 1984; Vann et al., 2007) (Fig. 1). These mammillary body efferents are particularly striking, as it appears that almost every neuron within the mammillary bodies projects to the anterior thalamic nuclei (Guillery, 1955; Vann et al., 2007; Aggleton et al., 2010). Yet, the separate functional significance of these mammillary body inputs to the anterior thalamic nuclei has often been overlooked (Vann, 2010).

Indeed, most accounts of mammillary body function, and by inference the mammillary body-anterior thalamic axis, again highlight the importance of hippocampal connections to this region, such that the mammillary bodies are often referred to as a constituent of an ‘extended hippocampal system’ that simply relay hippocampal inputs to the anterior thalamus (e.g. Aggleton and Brown, 1999; Delay and Brion, 1969; Gaffan, 1992). Apparent support for this position comes from evidence that, like the hippocampus and anterior thalamus, damage to the mammillary bodies and their thalamic projections can result in memory impairments in both humans and rodents (e.g. Gudden, 1896; Carlesimo et al., 2007; Van der Werf et al., 2003a,b; Vann and Aggleton, 2003; Yoneoka et al., 2004). The unidirectional nature of subicular complex inputs to the mammillary bodies, and thence to the anterior thalamic nuclei, might also appear to be consistent with the notion of an ‘extended hippocampal system’ (Aggleton et al., 2005). This account has two major shortcomings. First, it ascribes no independent role to the mammillary body-anterior thalamic axis, thereby effectively rendering it redundant and second, it completely overlooks the non-hippocampal inputs to the mammillary bodies that originate predominately in the limbic midbrain.

Recent advances in our understanding of the mammillary bodies and their thalamic projections challenge hippocampal-centric models of memory. By revealing a role for the mammillary bodies in mnemonic processes that is independent of its inputs from the subicular complex, this work heralds the need to look beyond the hippocampus and consider a wider network of structures that may contribute to mammillary body, and in turn anterior thalamic nuclei, function. These advances in our understanding of both the anatomical and functional properties of the mammillary bodies and the implications for diencephalic, and in particular anterior thalamic contributions to cognition, will be the focus of this review.

## 2 Anatomy

The mammillary bodies comprise two main subregions: the medial and lateral nuclei. In turn, the medial mammillary bodies can be further divided into pars lateralis and pars medialis. Although there are differences in cell morphology between the medial and lateral nuclei, there appears to be only one cell type in each nucleus (Veazey et al., 1982). All the cells in the mammillary bodies appear to be projections cells and there are no apparent interneurons (Takeuchi et al., 1985; Veazey et al., 1982).

### 2.1 Connectivity

In contrast to other structures within Papez’ circuit, the mammillary bodies have major connections with only a limited number of sites. As these connections are principally via major fiber tracts, selective disconnections of mammillary body inputs and outputs are possible. For example, transection of the mammillothalamic tract selectively disconnects mammillary body inputs to the anterior thalamic nuclei (see Fig. 1) and, thus, allows a direct assessment of mammillary body contributions to anterior thalamic function. It is beyond the scope of this current review to provide an exhaustive account of mammillary body anatomy (see Vann, 2010). Rather, we will focus on those aspects of mammillary body anatomy that are particularly germane to anterior thalamic function.

The principal direct inputs to the mammillary bodies are from the hippocampal formation via the descending component of the postcommissural fornix and from the tegmental nuclei of Gudden via the mammillary peduncle. In turn, the mammillary bodies project via the mammillothalamic tract to the anterior thalamic nuclei and project back to the tegmental nuclei of Gudden (via the mamillotegmental tract). The lateral and medial nuclei are connected to the same overall structures but, within those structures, each mammillary nuclei is connected with a different sub-region to form two parallel systems (Vann and Aggleton, 2004; Hopkins, 2005) (Fig. 1). In terms of the hippocampal formation, the medial mammillary nuclei are innervated by projections arising in the dorsal, ventral and intermediate subiculum and the medial entorhinal cortex, while the lateral mammillary body nuclei receive inputs from presubiculum, parasubiculum and postsubiculum (Allen and Hopkins, 1989; Shibata, 1988; Swanson and Cowan, 1977; Van Groen and Wyss, 1990; Wright et al., 2010). It is important to note that, although the anterior thalamus is also richly innervated by the subiculum, these connections largely arise from different populations of cells: subicular neurons projecting to the anterior thalamus originate in the deepest cell populations, while those projecting to the mammillary bodies are located more superficially (Ishizuka, 2001; Wright et al., 2010; Yoder and Taube (2011)). The implication, as yet untested, is that these different subicular neurons convey parallel but independent information to the mammillary bodies and anterior thalamic nuclei, respectively. The medial mammillary nuclei project ipsilaterally to the anteromedial and anteroventral thalamic nuclei, whereas the lateral mammillary body nuclei have bilateral projections to the anterodorsal thalamic nuclei (Cruce, 1975; Seki and Zyo, 1984; Vann et al., 2007). Midbrain tegmental connections with the mammillary bodies follow the same parallel topography, as the medial mammillary nuclei have reciprocal connections with the ventral tegmental nuclei of Gudden, while the lateral mammillary nuclei exhibit reciprocal connections with the dorsal tegmental nucleus of Gudden (Cruce, 1977; Hayakawa and Zyo, 1984, 1985; Veazey et al., 1982). Afferent projections to both the medial and lateral nuclei arise in the supramammillary nucleus, the tuberomammillary nucleus and the septal region (Gozalo-Ruiz et al., 1992). In contrast to this pattern, only the medial mammillary body nuclei are innvervated by the prefrontal cortex (Allen and Hopkins, 1989) (Fig. 1).

While the afferent projections to the mammillary bodies are both excitatory and inhibitory, the principal efferent connections are solely excitatory. Inputs from both the hippocampal formation and the prefrontal cortex are excitatory but the projections from the tegmental nuclei are inhibitory (Allen and Hopkins, 1989). Mammillary body efferents to both anterior thalamic and tegmental nuclei are excitatory (Allen and Hopkins, 1990; Gozalo-Ruiz et al., 1998). Neurochemically, the efferents to the anterior thalamic nuclei use glutamate, aspartate and enkephalin (Gozalo-Ruiz et al., 1998).

### 2.2 Electrophysiological properties

Investigations into the electrophysiological properties of the mammillary bodies provide particularly informative insights into the importance of mammillary body efferents for anterior thalamic function. Consistent with the differential anatomical connectivity of the two main mammillary body sub-compartments, the lateral and medial mammillary nuclei have been shown to possess very different electrophysiological properties, which in turn make parallel, but distinct, contributions to the anterior thalamic nuclei.

#### 2.2.1 Lateral mammillary nucleus

**Fig. 2:**
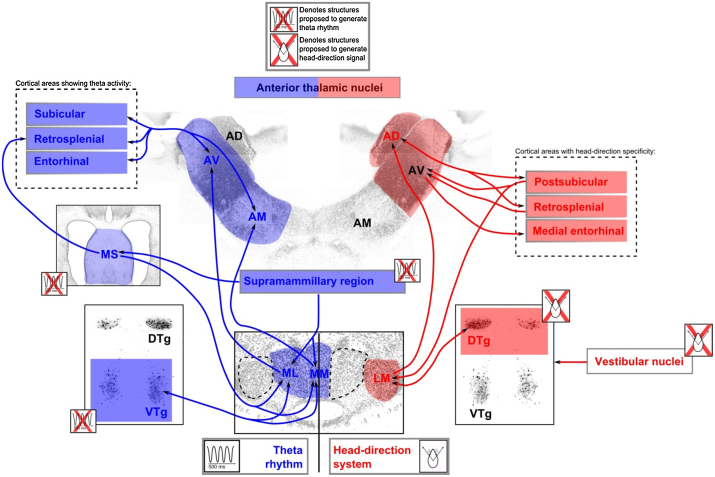
Semi-schematic diagram showing the neural connectivity that is thought to underlie theta (left; blue) and head-direction (HD; right; red) systems. Theta rhythm (blue): Cells within the medial mammillary nuclei (comprising pars medialis (MM) and pars lateralis (ML) subdivisions), as well as the anteroventral, (AV) and anteromedial (AM) thalamic nuclei exhibit oscillations at a frequency of theta (4–10 Hz). Head-direction system (red): Dorsal tegmental nucleus of Gudden → lateral mammillary body nuclei (LM) → anterodorsal thalamic nuclei (AD) connectivity is thought to underlie the HD and angular velocity systems. (For interpretation of the references to color in this figure legend, the reader is referred to the web version of this article.)

Both head-direction and angular velocity cells have been found in the lateral mammillary nuclei (Blair et al., 1998; Stackmam and Taube, 1998). Head-direction cells fire preferentially as a function of directional heading in the horizontal plane, while angular velocity cells discharge depending on the velocity of head movements (Taube et al., 1990a; Stackmam and Taube, 1998; Taube, 2007). The lateral mammillary nuclei require inputs from the dorsal tegmental nucleus of Gudden to generate head-direction signal (Bassett et al., 2007). Moreover, through its connection with other brain regions, including the postsubiculum and the anterodorsal thalamic nuclei, the lateral mammillary nuclei are well-placed to moderate the head-direction signal throughout the head-direction circuit (Fig. 2). Indeed, the importance of inputs from the lateral mammillary body nuclei to the anterior thalamic nuclei for the generation of head-direction cell activity is demonstrated by the finding that lesions to the lateral mammillary body nuclei completely abolish the head–direction signal in the anterodorsal thalamic nuclei (Blair et al., 1998, 1999; Bassett et al., 2007). In turn, the head-direction activity in the postsubiculum depends upon inputs from the anterodorsal thalamic nuclei (Goodridge and Taube, 1997).

#### 2.2.2 Medial mammillary nucleus

Nearly all cells in the medial mammillary nuclei modulate their firing rate at a frequency of theta (Bland et al., 1995; Kirk et al., 1996; Kocsis and Vertes, 1994). Through its temporal encoding and decoding of neuronal ensembles and the modification of synaptic weights, theta rhythm within hippocampal-diencephalic circuits is thought to be engaged in processes that are critical to memory formation (Buzsáki, 2002, 2005; Rutishauser et al., 2010). A fundamental understanding of what drives theta rhythm within this circuit is, however, still elusive. One view holds that theta activity in the medial mammillary nuclei is driven by descending projections from the hippocampal formation. Support for this position comes from the finding that there is a strong correlation between the onset and rate of mammillary body and hippocampal theta oscillations (Kocsis and Vertes, 1994) as well as the demonstration that inactivation of the medial septum attenuates the theta rhythm in both the hippocampus and mammillary bodies (Kirk et al., 1996) (Fig. 2). Similarly, recent evidence has shown that hippocampal, but not mammillary body inputs, moderate theta-related plasticity in the anterior thalamus, as the amplitude of anterior thalamic theta spectral power is selectivity increased by low frequency stimulation of fornical-anterior thalamic inputs (Tsanov et al., 2011a). An alternative view holds that the ventral tegmental nucleus of Gudden, which is reciprocally connected with the medial mammillary nuclei, may modulate theta within this circuit (Kocsis et al., 2001; Vertes et al., 2004). All cells in the ventral tegmental nucleus of Gudden fire rhythmically with theta (Kocsis et al., 2001) and, in fact, rhythmic-bursting recordings from this structure occur 1–2 s before the onset of hippocampal theta (Bassant and Poindessous-Jazat, 2001). The suggestion that the ventral tegmental nucleus of Gudden may mediate hippocampal theta activity via its mammillary body connections is partially supported by findings that extensive electrolytic lesions to the supramammillary nucleus, and adjacent mammillary bodies, attenuate both the frequency and amplitude of theta mediation of cell firing in the hippocampus (Sharp and Koester, 2008; Thinschmidt et al., 1995). However, as the lesions in these studies involved almost the entire mammillary body region, the neuroanatomical locus of these effects remains to be elucidated. A challenge for future studies will be to selectively target the different components of this region, in order to ascertain if the mammillary bodies have any specific involvement in the regulation of theta activity within the hippocampal-diencephalic network.

There is, however, good evidence that learning-induced plasticity within the anterior thalamus depends on excitatory inputs from the mammillary bodies. For example, when rabbits learn a conditional avoidance discrimination, projections from the mammillary bodies are necessary for behaviour-related activity changes in the anteroventral thalamic nucleus (Gabriel et al., 1995). Moreover, projections carried in the mammillothalamic tract also support spontaneous baseline unit activity in the anteroventral thalamic nuclei (Gabriel et al., 1995). Comparisons of activity-dependent plasticity within the anterior thalamus after stimulation of either the dorsal fornix or the mammillothalamic tract have confirmed the importance of mammillary body afferents for plasticity within the anterior thalamic nuclei (Tsanov et al., 2011b). High-frequency stimulation of the mammillothalamic tract induces large-amplitude and stable long-term-potentiation of the anterior thalamic field response, which is not reproduced by equivalent stimulation of the dorsal fornix (Tsanov et al., 2011b). In contrast, low-frequency stimulation of the mammillothalamic tract does not evoke depression in the anterior thalamic field response, whereas long-term-depression is induced by low-frequency stimulation of hippocampal projections (Tsanov et al., 2011b). These data imply a tendency for mammillothalamic and fornical inputs to the anterior thalamus to oppose one another, i.e. mammillothalamic inputs elevate, while fornical inputs attenuate the polarity of anterior thalamic plasticity. Furthermore, this same study found activation-dependent, brain-derived neurotrophic factor (BDNF)-mediated augmentation of basal synaptic transmission modulated by the mammillothalamic tract (Tsanov et al., 2011b). More broadly, such findings suggest that mammillary body and hippocampal inputs make complementary, rather than overlapping, contributions to anterior thalamic function.

## 3 Mammillary body function

### 3.1 Clinical studies

Although the mammillary bodies, and their projections to the anterior thalamus, have long been assumed to be important for episodic memory in humans (e.g. Gudden, 1896), it has only been until relatively recently that their involvement has been confirmed empirically. For example, the neuropathology of Korsakoff's syndrome was first described over a century ago but the neuroanatomical locus of this syndrome has not been clearly established in the intervening years. It is, however, now apparent that damage to both the mammillary bodies and the mammillothalamic tract can contribute to this distinctive clinical condition (Kril and Harper, 2012; Gold and Squire, 2006; Yoneoka et al., 2004). A notable finding is the demonstration that damage to the mammillothalamic tract is the sole consistent predictor of anterograde amnesia following a thalamic stroke (Carlesimo et al., 2011; Clarke et al., 1994; Van der Werf et al., 2000, 2003a,b). A further study also revealed the mammillary bodies to be the only site consistently linked to recollective memory impairments in patients who had undergone surgery for the removal of colloid cysts (Tsivilis et al., 2008; Vann et al., 2009). The patients, matched on all factors other than the degree of mammillary body atrophy, differed significantly on measures of recollection, but not familiarity-based recognition (Tsivilis et al., 2008; Vann et al., 2009). Although severance of the fornix is known to result in amnesia (e.g. Gaffan and Gaffan, 1991), in the Tsivilis et al. (2008) study, neither fornix nor parahippocampal cortex measures were consistently associated with recall performance. The importance of the mammillary bodies for episodic memory is further revealed by findings from the only known case of a patient with pathology restricted to the mammillary bodies. Patient B.J., who suffered selective bilateral damage to the mammillary bodies after a snooker cue was forced up his nose, experienced relatively mild anterograde amnesia but spared recognition memory (Dusoir et al., 1990). A loss of verbal long-term memory was also reported in N.A., a patient who suffered unilateral diencephalic damage which included the mammillothalamic tract (Teuber et al., 1968). This dissociation between impaired recollection and spared familiarity recognition memory was also found in an amnesic patient, G.P, who sustained bilateral mammillothalamic damage (Carlesimo et al., 2007).

Although damage to thalamic nuclei almost certainly contributes to diencephalic amnesia, these studies highlight the paramount importance of mammillary body projections to the anterior thalamic nuclei for recollective but not familiarity-based memory (Aggleton et al., 2011). Nonetheless, the difficulty of finding cases with circumscribed damage within this circuit limits the extent to which definitive conclusions can be drawn from these clinical cases. Similarly, the value of current functional imaging techniques for investigating mammillary body and anterior thalamic function in humans is constrained by both the size and position of these structures. These observations underscore the need for animal models.

### 3.2 Animal models

#### 3.2.1 Behavioural lesion studies

As most current models of mammillary body function emphasise the importance of their inputs from the hippocampal formation, the overwhelming majority of behavioural studies in rodents have focused on spatial memory (Vann, 2010). For the very same reason, most studies evaluating the behavioural effects of anterior thalamic damage in rodents also tend to assay spatial memory (Jankowski et al., 2013). This almost singular focus on spatial memory has the advantage that it allows direct comparisons between the effects of lesions targeted at these two brain sites on the same behavioural tasks, but it does potentially overlook additional contributions that the mammillary bodies might be making to anterior thalamic function. When evaluating mammillary body contributions to anterior thalamic function, lesions of the mammillothalamic tract are particularly informative as they provide a direct assessment of the functional significance of mammillary body inputs to the anterior thalamic nuclei.

Lesions of the mammillary bodies impair performance on tests of both reinforced and spontaneous T-maze alternation (Aggleton et al., 1995; Béracochéa and Jaffard, 1987, 1990, 1995; Gaffan et al., 2001; Rosenstock et al., 1977; Vann and Aggleton, 2003). These deficits are found on both the standard and continuous alternation variants of the task (Aggleton et al., 1995; Field et al., 1978; Vann and Aggleton, 2003). Performance on a further test of spatial working memory, the radial-arm maze task, has also been shown to be sensitive to the effects of mammillary body lesions (Jarrad et al., 1984; Neave et al., 1997, Sziklas and Petrides, 1993; Vann and Aggleton, 2003). The radial-arm maze task requires animals to retrieve rewards from the arms of the maze without re-entering the same arm; effective performance requires the animal to monitor the arms it has already entered. Transection of the mammillothalamic tract reliably produces equivalent deficits to mammillary body lesions on both of these tests of spatial working memory (Field et al., 1978; Nelson and Vann, 2014; Vann and Aggleton, 2003; Vann, 2013). The effects of mammillary body lesions on spatial learning in the water-maze have also been examined. Although mammillary body lesions have been shown to both disrupt (Sutherland and Rodriguez, 1989) and spare reference memory (Santín et al., 1999), reliable and enduring impairments are found on delayed matching-to-place in the water-maze (Santín et al., 1999; Vann and Aggleton, 2003). Lesions to the mammillothalamic tract reproduce the effects of mammillary body lesions on delayed matching-to-place in the water-maze (Vann and Aggleton, 2003).

As tests of spatial working memory can potentially be solved by a variety of different strategies, studies that have sought to isolate the different strategies available to the animal can be particularly informative about the nature of the spatial learning deficit associated with mammillary body and mammillothalamic tract lesions. One plausible suggestion is that impaired navigation could provide a unifying account of the pattern of results obtained (e.g. Winter et al., 2011). However, this account seems unlikely as mammillary body and mammillothalamic tract lesions do not always lead to a spatial learning deficit, even on tasks where the animal is required to navigate through the environment. For example, mammillothalamic tract lesions do not disrupt geometric learning in the water-maze (using the shape of the maze to find the hidden platform) (Vann, 2013) and reference memory in the water-maze can be unaffected by mammillary body lesions (Santín et al., 1999). Conversely, lesion deficits emerge on tasks which contain little or no navigational component (Nelson and Vann, 2014). For example, mammillothalamic tract lesions disrupt the ability to use distal visual cues to discriminate between two locations within a room, irrespective of the direction travelled (Nelson and Vann, 2014). Rather, it would appear that mammillary body and mammillothalamic tract lesions produce deficits when animals are required to use distal spatial cues or the rapid encoding of new spatial information is required. For example, in the radial-arm maze task the most pronounced deficits emerge when the maze is rotated mid-way through the session such that the value of intra-maze cues are nullified and animals are forced to rely on extra-maze cues (e.g. Nelson and Vann, 2014; Vann and Aggleton, 2003; Vann, 2013). Similar results have been found in the T-maze when the sample and choice phases are run in separate mazes so that the animal cannot use intra-maze cues to alternate (Vann, 2013). This account would also explain why delayed non-matching-to-place but not reference memory (e.g. Santín et al., 1999; Vann and Aggleton, 2003) is particularly sensitive to mammillary body and mammillothalamic tract lesions, as the former task places a premium on the rapid encoding of new spatial information.

The effects of mammillary body lesion are not restricted to tasks that involve navigation. Both mammillary body and mammillothalamic tract lesions have been shown to disrupt certain forms of contextual learning. Vann et al. (2003) showed that mammillothalamic tract lesions retarded the acquisition of a visuo-spatial, but not a non-spatial, contextual discrimination in which animals are required to respond differentially to stimuli depending on the context. Similarly, mammillary body lesions in mice disrupt contextual fear conditioning but spare cued fear conditioning (Celerier et al., 2004). However, mammillothalamic tract lesions impair discriminative avoidance behaviour in rabbits; lesion animals were slower to learn an avoidance response to a tone predictive of shock (Gabriel et al., 1995). Although mammillary body and mammillothalamic tract lesions do not disrupt rats’ ability to recognise a novel from a familiar item (Aggleton et al., 1995) recent evidence has shown that mammillothalamic tract lesions do impair object-in-place memory (Nelson and Vann, 2014). Object-in-place memory does not tax recognition memory per se but rather requires the animal to link a specific object with a specific location. Similarly, mammillary body lesions in monkeys spare recognition memory for objects (Aggleton and Mishkin, 1985) but impair the ability to learn object-in-place scenes (Parker and Gaffan, 1997a). This apparent functional dissociation between object recognition and object-in-place memory mirrors the aforementioned clinical evidence showing that pathology of the mammillary body or mammillothalamic tract impairs recollection but spares familiarity on tests of recognition memory (e.g. Carlesimo et al., 2007; Tsivilis et al., 2008; Vann et al., 2009) as well as the finding of anterograde amnesia with spared recognition memory in patient B.J. (Dusoir et al., 1990).

A challenge for future studies will be to examine whether the mammillary bodies have a broader role in learning and memory beyond the spatial domain. Evidence from patient studies certainly points to such a role, but currently, there is a paucity of data from animal models on non-spatial functions. Nevertheless, there is an emerging appreciation that the anterior thalamic nuclei may also support non-spatial functions. For example, evidence has highlighted anterior thalamic involvement in certain forms of recency judgements (Wolff et al., 2006; Dumont and Aggleton, 2013) but it is currently unclear the extent to which these effects depend on inputs from the mammillary bodies.

#### 3.2.2 Lateral versus medial mammillary nucleus lesions

Given the aforementioned anatomical and electrophysiological data, it also important to consider any differential effects of medial versus lateral mammillary nucleus lesions on behaviour, as the results from these studies may provide insights into the potentially distinct contributions that these separate nuclei make to anterior thalamic function (Vann and Aggleton, 2004; Vann, 2010). That said, the overwhelming majority of lesion studies either involved the medial mammillary nuclei (e.g. Béracochéa and Jaffard, 1987, 1995; Field et al., 1978; Rosenstock et al., 1977; Santín et al., 1999) or included almost the entire mammillary body region (e.g. Tonkiss and Rawlins, 1992; Sziklas and Petrides, 2000). Furthermore, mammillothalamic tract lesions may selectively disconnect medial mammillary nucleus projections to the anterior thalamus but spare those from the lateral mammillary nuclei (Vann and Albasser, 2009). Thus, there remains a paucity of data describing the behavioural effects of selective lateral mammillary nucleus lesions. From the two studies that have selectively targeted these nuclei, it seems that lateral mammillary nucleus lesions produce a far less profound deficit on tests of spatial memory than is apparent following complete mammillary body lesions. For example, lateral mammillary nucleus lesions spare the initial acquisition of T-maze alternation (Vann, 2005) and mild deficits only emerge when the use of intra-maze cues is precluded by running the sample and choice phases in separate mazes (Vann, 2011). Similarly, and again in contrast to the effects of complete mammillary body lesions, working memory in the water maze is only transiently affected by lateral mammillary nucleus lesions (Vann, 2005). Conversely, lateral mammillary nucleus lesions retard the acquisition of a geometric task in the water maze (Vann, 2011) that has subsequently been shown to be unaffected by mammillothalamic tract lesions (Vann, 2013). This latter dissociation presumably reflects the fact that this geometric task may engage the head-direction system within which the lateral mammillary nuclei occupy a pivotal position (Taube et al., 1990b; Aggleton et al., 2009; Vann, 2011) (Fig. 2). As the impact of lateral mammillary nucleus lesions does not reproduce the effects of complete mammillary body damage, it would seem that the pattern of spatial memory impairments seen after complete mammillary body lesions cannot solely be ascribed to a loss of head-direction information. The further implication is that the medial mammillary body nuclei make additional contributions to spatial memory.

More broadly, these results accord with the proposition that these two mammillary nuclei make quantifiably different contributions to mnemonic processes (Vann and Aggleton, 2004; Hopkins, 2005). This dissociation, in turn, maps onto proposed functional differences in the projection targets of the lateral and medial mammillary nuclei. The anterodorsal thalamic nuclei, directly innervated by the lateral mammillary nuclei, form a key node within the head-direction system. The medial mammillary nuclei project to both anteromedial and anteroventral thalamic nuclei: The anteromedial thalamic nuclei are thought to be important for relaying information from the hippocampal-diencephalic circuit to prefrontal areas, while it has been suggested that the anteroventral thalamic nuclei convey theta-activity to the hippocampal formation (Aggleton et al., 2010; Jankowski et al., 2013).

### 3.3 Comparison of mammillary body and anterior thalamic lesion effects

Anterior thalamic nucleus lesions produce deficits on the same tests of spatial memory as mammillary body lesions, consistent with both structures forming part of an extended memory network. Working memory assessed in both the T-maze and eight arm radial-maze has been shown to be disrupted by lesions of the anterior thalamus (e.g. Aggleton et al., 1995, 1996; Byatt and Dalrymple-Alford, 1996; Mair et al., 2003; Mitchell and Dalrymple-Alford, 2005, 2006; Warburton et al., 1997, 1999). Similarly, anterior thalamic damage results in impairments on both reference memory and delayed-non-matching to sample in the water-maze (e.g. Sutherland and Rodriguez, 1989; Van Groen et al., 2002; Warburton et al., 1999; Warburton and Aggleton, 1999). Lesions centred on the anterodorsal thalamic nuclei also disrupt the same geometric task that is sensitive to lateral but not medial mammillary nucleus lesions (Aggleton et al., 2009; Vann, 2011, 2013), consistent with this task being supported by the lateral mammillary nucleus-anterodorsal thalamic nuclei projections. Although few studies have directly compared the effects of mammillary body and anterior thalamic nuclei lesions on tests of spatial memory, there is some evidence that anterior thalamic lesions may produce more severe deficits than either mammillary body or mammillothalamic tract lesions (e.g. Aggleton et al., 1991, 1995). For example, lesions of the anterior thalamic nuclei lead to a robust and enduring T-maze alternation deficit, while mammillary body and mammillothalamic tract lesion impairments on this task can be ameliorated by extended training (e.g. Vann and Aggleton, 2003) or only emerge when animals are forced to rely on allocentric spatial information (e.g. Vann, 2013).

There are several possible explanations as to why disrupting mammillary body inputs to the anterior thalamus may not always reproduce exactly the effects of lesions to the anterior thalamic nuclei themselves. One is selectivity of the lesion: mammillothalamic tract lesions are usually highly selective (e.g. Vann and Aggleton, 2003; Vann, 2013), whereas the location of the anterior thalamic nuclei means that lesions to this structure often result in unintended damage to adjacent structures including the medial dorsal and laterodorsal thalamic nuclei, the intralaminar thalamic nuclei, the rhomboid nucleus and nucleus reuniens. The extent to which this damage may contribute to the pattern of deficits observed after anterior thalamic lesions is not entirely clear. It should be acknowledged that selective damage to these adjacent structures does not reproduce the effects of anterior thalamic lesions (e.g. Hunt and Aggleton, 1998; Loureiro et al., 2012; Wolff et al., 2008) and, where deficits have been reported (e.g. Savage et al., 1998), the lesions encroached on the anterior thalamic nuclei. Nevertheless, there is evidence that cell loss beyond the anterior thalamic nuclei may magnify spatial impairments, particularly when the lesions involve the lateral dorsal nucleus (Warburton et al., 1997, 1999). In this respect, it is noteworthy that mammillothalamic tract lesions do not disconnect the laterodorsal thalamic nuclei. A further, and perhaps more significant, consideration is anatomical: in the absence of their mammillary body inputs, the anterior thalamic nuclei are still directly innervated by the hippocampal formation. Given their distinct electrophysiological properties, fornical and mammillothalamic inputs to the anterior thalamus are unlikely to have duplicate functions (Tsanov et al., 2011a,b). Similarly, the anterior thalamic nuclei and the mammillary bodies receive inputs from distinct cell populations within the hippocampal formation (Ishizuka, 2001; Wright et al., 2010; Yoder and Taube, 2011). As such, direct hippocampal afferents may support different mnemonic processes that may allow a degree of functional compensation for the loss of the mammillary body inputs. Furthermore, other inputs to the anterior thalamic nuclei may support spatial cognition. For example, brainstem cholinergic innervation to the anteroventral thalamic nuclei is known to influence spatial memory (Mitchell et al., 2002). Multiple brain sites support spatial cognition and, therefore, structures beyond the medial diencephalon may also partially counteract the effects of mammillary body lesions. A related account holds that mammillary body or mammillothalamic tract lesions may only disrupt a subset of spatial processes (e.g. allocentric information). As tests of spatial memory can potentially be solved by a variety of strategies, other available classes of spatial information may be sufficient to support task performance so animals are able to switch to alternative strategies. For example, mammillary body and mammillothalamic tract lesions do not appear to preclude the use intra-maze cues in tests of spatial working memory (Vann and Aggleton, 2003; Vann, 2013).

As with mammillary body and mammillothalamic tract lesions, anterior thalamic nuclei lesion effects are also found on behavioural tasks that do not tax spatial navigation. The anterior thalamus does not appear to be necessary for certain aspects of recognition memory. It has been consistently shown that anterior thalamic nuclei lesions do not disrupt a rat's ability to recognise a novel from a familiar object (Moran and Dalrymple-Alford, 2003; Warburton and Aggleton, 1999; Wilton et al., 2001). Conversely, anterior thalamic lesions do disrupt object-in-place memory (Sziklas and Petrides, 1999; Wilton et al., 2001) as well as recency memory for objects and events (Dumont and Aggleton, 2013; Wolff et al., 2006). In monkeys, object-in-place memory is also disrupted by damage to the anterior thalamus (Parker and Gaffan, 1997b). This pattern of results accords with the proposition that the mammillary body-anterior thalamic axis plays a preponderant role in recollective rather than familiarity-based recognition memory (e.g. Aggleton et al., 2011). Similarly, there is evidence that the processing of contextual information is disrupted following anterior thalamic nuclei lesions (e.g. Law and Smith, 2012; Marchand et al., 2013; but see: Dumont et al., 2014; Dupire et al., 2013).

## 4 Which inputs are driving mammillary body function?

Most accounts of anterior thalamic function stress the importance of their hippocampal inputs (e.g. Aggleton and Brown, 1999; Papez, 1937; Delay and Brion, 1969). Evidence in support of this position is threefold. First, the anterior thalamic nuclei receive both direct, via the fornix, and indirect, via the mammillary bodies, inputs from the subicular complex; second, lesions to both the anterior thalamic nuclei and mammillary bodies produce almost comparable deficits on tests of spatial cognition and third, disconnection studies have confirmed the importance of interactions between the anterior thalamic nuclei and hippocampus for spatial learning (e.g. Henry et al., 2004; Warburton et al., 2001). The implication of this account is that the hippocampal formation acts upon the anterior thalamus through these parallel routes, and it is the loss of this information flow that accounts for the spatial deficits observed after mammillary body damage. In this scenario, the mammillary bodies make no independent contribution to anterior thalamic function and simply act as a relay for hippocampal information.

**Fig. 3:**
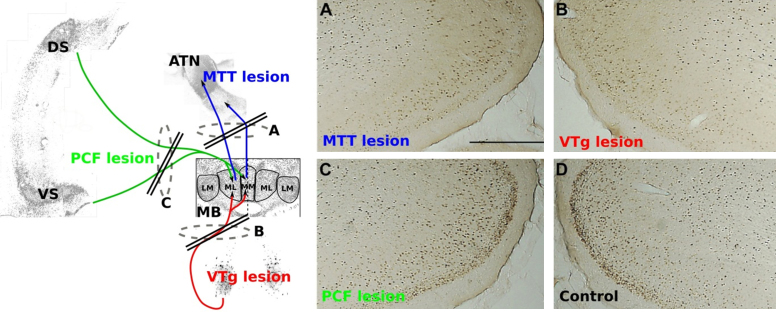
Photomicrographs of c-Fos immunoreactive cells in the retrosplenial cortex following: (A) mammillothalamic tract lesion (MTT; blue); (B) ventral tegmental nucleus of Gudden lesion (VTg; red); (C) descending postcommissural fornix lesion (PCF; green); (D) surgical control. To the left is a semi-schematic representation of the corresponding tract disconnections. *Abbreviations*: ATN, anterior thalamic nuclei; DS, dorsal subiculum; LM, lateral mammillary nuclei; MB, mammillary bodies, ML, medial mammillary nucleus, pars lateralis; MM, medial mammillary nucleus, pars medialis; VS, ventral subiculum; VTg, ventral tegmental nucleus of Gudden. (Modified, with permission from Vann, 2013). (For interpretation of the references to color in this figure legend, the reader is referred to the web version of this article.)

**Fig. 4:**
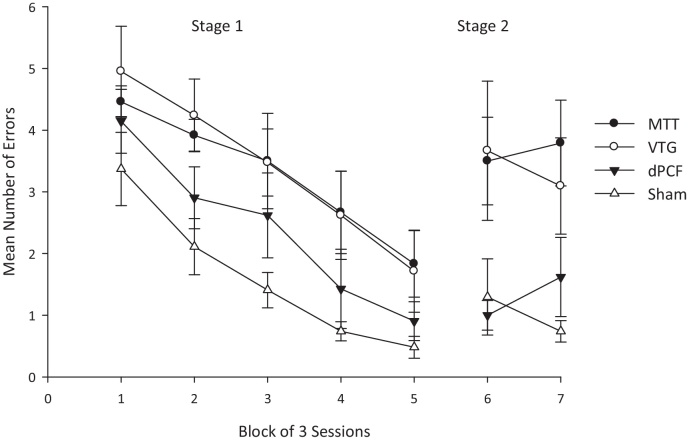
Comparison of the effects of lesions to the mammillothalamic tract (MTT), the ventral tegmental nuclei of Gudden (VTG) or the descending postcommisural fornix (dPCF) and control animals (Sham) on spatial working memory in the radial arm maze. The graph shows the mean number of errors in blocks of 3 sessions on rats performing the standard working memory task (stage 1). In stage 2, to nullify the value of intramaze cues, the maze was rotated half-way through the session. In Stage 1, the MTT and VTG groups were significantly impaired relative to the shams. In Stage 2 when the maze was rotated, the MTT and VTG groups were impaired relative to both the Sham and dPCF groups. Performance in the Sham and dPCF did not differ in either stage. Adapted from Vann (2013).

A prediction that follows from this account is that disconnection of the descending hippocampal projections to the mammillary bodies (Fig. 3) should not only disrupt spatial learning, but should result in impairments that are equivalent to those seen after mammillary body and mammillothalamic tract damage. However, neither of these predictions is supported by empirical findings. Transection of the descending postcommissural fornix, which selectively disconnects the hippocampal inputs to both the medial and lateral mammillary nuclei, appears to have minimal discernible impact on tests of spatial memory. These lesions produce only a borderline T-maze alternation deficit and spare acquisition of both the radial-arm maze and water-maze working memory tasks (Vann et al., 2011). This pattern of results stands in stark contrast to the marked deficits observed after mammillary body and mammillothalamic tract damage (e.g. Vann and Aggleton, 2003). Indeed, Vann (2013) directly compared the effects of mammillothalamic tract and descending postcommissural fornix lesions on various behavioural assays of spatial memory and the results were clear: lesions of the mammillothalamic tract, but not of the descending postcommissural fornix, led to consistent spatial memory deficits (Fig. 4). Of course, it could be argued that the lack of a descending postcommissural fornix lesion effect on these tests simply reflects functional compensation for the loss of the indirect inputs by the direct hippocampal projections to the anterior thalamic nuclei. Two lines of evidence militate against such an explanation. If the direct hippocampal projections to the anterior thalamic nuclei are able to compensate for the loss of the indirect pathway, then this compensation should be equally evident after damage to the mammillary bodies or the mammillothalamic tract (as these lesions only disconnect the indirect pathway). According to this account, all three lesions (mammillary body, mammillothalamic tract and descending postcommissural fornix) should have equivalent effects on tests of spatial memory. Yet, mammillary body and mammillothalamic tract lesions produce consistent spatial memory impairments that are markedly more severe than the deficits found after descending postcommissural fornix lesions (e.g. Vann and Aggleton, 2003; Vann et al., 2011; Vann, 2013). Furthermore, the two pathways, far from functioning in concert, have antagonistic electrophysiological properties (Tsanov et al., 2011b) that make compensation seem unlikely. The implications of these observations for mammillary body and anterior thalamic function are manifest. First, the mammillary bodies are able to support spatial cognition in the absence of their subicular complex inputs and so make a contribution to memory that is independent of the hippocampal formation. Second, mammillary body, and not the indirect hippocampal, inputs are vital to understanding anterior thalamic function. More broadly, they herald the functional importance of other, non-hippocampal, inputs to the mammillary body-anterior thalamic nuclei axis.

Obvious candidates are the dense connections that arise in the limbic mesencephalon: the medial mammillary body nuclei are innervated by the ventral tegmental nuclei of Gudden and the lateral mammillary body nuclei by the dorsal tegmental nuclei of Gudden. It is possible that these distinct nuclei are a pivotal source of inputs that support spatial processes within the medial and lateral mammillary nuclei, respectively. Until recently, little was known about the functional significance of these inputs to the mammillary bodies. However, it is now evident that selective lesions to the ventral tegmental nucleus of Gudden produce clear impairments on an array of spatial memory tasks, which are known to be sensitive to both mammillary body and anterior thalamic damage, including working memory in the water-maze and radial arm-maze as well as reinforced alternation in the T-maze (Vann, 2009). Not only do mammillothalamic tract and ventral tegmental nucleus of Gudden lesions produce equivalent impairments on these tasks, but both surgeries lead to more enduring and robust deficits than lesions of the descending postcommissural fornix (Vann, 2013). This pattern of results suggests that the ventral tegmental nucleus of Gudden is able to maintain mammillary body function in the absence of their inputs from the descending postcommissural fornix, i.e. hippocampal formation. The implication is that information streams from the ventral tegmental nucleus of Gudden make distinct contributions to mammillary body-anterior thalamic nuclei function that are vital for spatial memory. Indeed, the report of a patient who had become amnesic following damage in the region of the ventral tegmental nucleus of Gudden is consistent with the importance of this nucleus for memory (Goldberg et al., 1981). Further evidence in support of the significance of this pathway comes from an examination of the impact of lesions within this network on markers of plasticity in other related brain sites. It is well-documented that lesions to the anterior thalamic nuclei result in hypoactivity, as indexed by the expression of immediate-early genes, such as c-*fos*, in an array of distal brain regions including the hippocampus, the retrosplenial cortex and the prefrontal cortex (for a review see Aggleton and Nelson, current issue). A remarkably similar pattern of hypoactivity is also observed following mammillothalamic tract transection (Vann and Albasser, 2009) (Fig. 3). The qualitative and quantitative equivalence of anterior thalamic and mammillothalamic tract lesion effects on the pattern of dysfunction observed in distal regions raises the possibility that these anterior thalamic lesion effects are principally driven by the loss of their mammillary body inputs. A further intriguing question is whether the observed hypoactivity following mammillothalamic tract lesions reflects the loss of indirect hippocampal inputs or the disconnection of afferents from the ventral tegmental nucleus of Gudden. If ventral tegmental nucleus of Gudden, and not hippocampal, inputs are critical for maintaining mammillary body function then lesions to the ventral tegmental nucleus of Gudden would also be expected to disrupt functional markers in the same distal brain regions. This prediction is borne out by empirical findings: ventral tegmental nucleus of Gudden and mammillothalamic tract lesions induce comparable reductions in c-Fos, positive cell counts in the same network of related structures (Vann, 2013) (Fig. 3). This cascade of pathological changes in distal sites is, however, not present after descending postcommissural fornix lesions (Vann, 2013). The implication of these results is that mammillothalamic tract lesion effects on markers of plasticity in distal brain regions are modulated by inputs to the medial mammillary nuclei from the limbic midbrain rather than the hippocampus. The convergence of evidence from these behavioural and imaging studies indicates that information flow from the ventral tegmental nucleus of Gudden is crucial to medial mammillary nuclei and, in turn, anterior thalamic function.

As noted previously, medial and lateral mammillary nuclei can be dissociated in terms of both function and connectivity. The lateral mammillary nuclei form a vital node within the head-direction system and this is reflected in their distinct connectivity, i.e. they have connections with the dorsal tegmental nucleus of Gudden, and in turn project to the anterodorsal thalamic nuclei (Fig. 2). Evidence from lesion and electrophysiological studies indicates that the head-direction signal is generated by the reciprocal connections between the dorsal tegmental nucleus of Gudden and the lateral mammillary nuclei (Bassett et al., 2007; Blair et al., 1998; Taube, 2007). Indeed, the integrity of this circuit appears to be crucial for maintaining the head-direction signal within the anterodorsal thalamic nuclei, as lesions to both structures abolish the head-direction signal in the anterodorsal thalamic nuclei (Bassett et al., 2007; Blair et al., 1998). Moreover, the importance of these connections is further highlighted by the apparent hierarchical organisation of the head-direction circuit: lesions to subcortical structures (e.g. dorsal tegmental nucleus of Gudden and lateral mammillary nuclei) abolish the head-direction signal in ‘higher’ components of the network (e.g. anterodorsal thalamic nuclei, postsubiculum) but damage to cortical sites does not disrupt the head-direction signal ‘lower’ down in the circuit (e.g. Clark and Taube, 2011). Further support for this proposition comes from recent behavioural findings that have shown that lesions to the dorsal tegmental nucleus of Gudden produce deficits in the acquisition of tasks in which directional heading is required (e.g. Dwyer et al., 2013; Frohardt et al., 2006). Dorsal tegmental nucleus of Gudden lesions do not just disrupt directional navigation but also lead to persistent impairments in place learning (Clark et al., 2013). Such findings accord with the emerging appreciation of the importance of the head-direction system for both directional and place navigation (e.g. Gibson et al., 2013). The extent to which hippocampal place cell activity depends on the signal from the head-direction system remains to be fully elucidated but consistent with this proposition, lesions within the head-direction system can disrupt the stability of hippocampal place cell firing (Calton et al., 2003). These findings highlight the potential contribution of the lateral mammillary nuclei and their inputs from the limbic midbrain to the processing of spatial information within the hippocampal place cell network.

## 5 Implications for anterior thalamic nucleus function

Traditional models of anterior thalamic function have stressed the importance of the direct and indirect (via the mammillary bodies) hippocampal inputs (e.g. Delay and Brion, 1969; Aggleton and Brown, 1999). These models effectively reduced the mammillary bodies to the status of a hippocampal relay and, thereby, afforded the mammillary bodies no independent role in mnemonic processes. More broadly, these models placed the hippocampus at the centre of the network of structures that support memory and overlooked the extent to which the medial diencephalon may reciprocally act upon the hippocampal formation. In light of recent empirical findings, such a position requires revision. It is now clear that the mammillary bodies not only contribute to memory, but that this contribution is largely independent of its hippocampal inputs. Rather, information streams from the limbic mesencephalon would appear to be vital to maintaining mammillary body function. As the mammillary bodies comprise two distinct subdivisions that can be dissociated both in terms of their hodology and function, there are at least two possible routes through which the mammillary bodies and their limbic midbrain afferents can influence anterior thalamic function. First, there is now considerable evidence that the dorsal tegmental nucleus of Gudden → lateral mammillary nuclei → anterodorsal thalamic nuclei pathway plays a critical role in both the generation and propagation of the head-direction signal (Fig. 2). Second, while the function of the ventral tegmental nucleus of Gudden → medial mammillary nuclei → anteroventral thalamic nuclei pathway is, at present, less clear, it is likely to include the regulation of theta rhythm and the optimisation of synaptic plasticity (Fig. 2). That the convergence on the anterior thalamic nuclei of these parallel but distinct information flows is required for normal memory is confirmed by lesions studies that have systematically disconnected the different components of these two systems (e.g. Bassett et al., 2007; Blair et al., 1998; Clark et al., 2013; Vann and Aggleton, 2003; Vann et al., 2011; Vann, 2009, 2013). How these different information streams are integrated to support memory is still open to conjecture but interactions between the head-direction signal and theta are likely to be significant (Aggleton et al., 2010; Jankowski et al., 2013). In this respect, a key discovery is the description of theta-modulated head- direction cells in the rat anteroventral thalamic nuclei that appear to integrate heading and movement information (Tsanov et al., 2011c). A further consideration is the role of medial mammillary body nuclei inputs to the anteromedial thalamic nuclei. The anteromedial thalamic nuclei stand out from the other thalamic nuclei in that they contain few theta-cells (6%), have only limited projections to the hippocampal formation but instead have strong reciprocal projections with an array of cortical sites including the anterior cingulate and prelimbic cortices (e.g. Albo et al., 2003; Shibata and Kato, 1993; Van Groen et al., 1999). Based on these properties, it has been posited that the anteromedial thalamic nuclei relay hippocampal-diencephalic signals to the prefrontal cortex and, in turn, support cognitive flexibility and other higher-order functions (e.g. Aggleton et al., 2010). As the medial mammillary nuclei receive excitatory inputs from the prefrontal cortex (Allen and Hopkins, 1989), they are well placed to influence such functions and may form part of a reciprocal loop between the medial diencephalon and the prefrontal cortex. This proposal is, as yet, untested but the implication is that there are other, perhaps non-spatial, medial mammillary body nuclei functions to be uncovered. Indirect support for this proposition comes from the recent finding that craniopharyngioma patients with hypothalamic injury, involving the mammillary bodies, show abnormal patterns of activation and deactivation in the prefrontal cortex, consistent with less efficient processing in a brain region engaged in executive functions (Özyurt et al., 2014).

## 6 Conclusions and remaining questions

This review set out to explain the significance of the mammillary body inputs for anterior thalamic function. Central to this process has been the specification of at least two parallel but separate routes through which the mammillary bodies can influence anterior thalamic function. Based on anatomical, electrophysiological and behavioural data, it is suggested that the mammillary bodies play an important role in supporting spatial memory through the propagation of both the head-direction signal and theta activity to the anterior thalamus. Significantly, this contribution appears to be largely independent of the hippocampus. Rather, inputs from the limbic midbrain appear critical to sustaining mammillary body function. Allied to this, recent evidence has suggested that the anterior thalamus does not simply relay incoming information, but actively integrates and modulates hippocampal-diencephalic information streams that are critical for mnemonic processes (e.g. Tsanov et al., 2011b–d). The implication of these findings is that the anterior thalamus may form a functional nexus that integrates limbic midbrain-diencephalic-hippocampal pathways to support mnemonic processes. In this scenario, the role of the mammillary bodies may well be to modulate these different information streams. As such, these findings challenge previous models that stressed the importance of the hippocampus for medial diencephalon function and highlight the need to consider how the medial diencephalon may act upon the hippocampal formation to support memory (Vann, 2009, 2010; Vann and Albasser, 2011).

This review has emphasised the importance of inputs from Gudden's tegmental nuclei but other sites connected with the mammillary bodies may also prove vital in understanding mammillary body and, more broadly, medial diencephalic function. While it is now apparent that the mammillary bodies are able to support spatial memory in the absence of information flow from the hippocampus, it seems unlikely that these inputs are redundant. Thus, one key challenge will be to elucidate the properties of the subicular inputs to the mammillary bodies and to contrast their function with the hippocampal projections to the anterior thalamus. Similarly, the status of the prefrontal afferents to the mammillary bodies is currently poorly understood but, in conjunction with the anteromedial thalamic nuclei, they may support non-spatial functions. Furthermore, interactions within local circuits between the mammillary bodies and the supramammillary nuclei are potentially critical to theta activity. Unravelling how these different pathways make seemingly separate but, at the same time, interdependent contributions to medial diencephalon function remains a key step towards understanding the wider neural circuitry that underpins memory.
